# Impact of chronotype on work-related quality of life in healthcare professionals: a cross-sectional study

**DOI:** 10.1192/j.eurpsy.2026.11717

**Published:** 2026-07-31

**Authors:** M. S. Majoul, L. Ben Hmida, L. S. Chaibi, M. Messelmani, Z. Moatemri, J. Zaouali

**Affiliations:** 1 Neurology; 2 pulmonology; 3 psychiatry, Military hospital of Tunis, Tunis, Tunisia

## Abstract

**Introduction:**

The impact of chronotype on alertness and performance at work in healthcare professionals (HPs) has been hypothesized. The relation between chronotype and work-related quality of life (WRQOL) remains to be investigated.

**Objectives:**

Our aim was to investigate the impact of the chronotype on WRQOL in HPs.

**Methods:**

A cross-sectional, monocentric, observational study was conducted at the military hospital of Tunis in September 2025, including HPs (nurses, nursing assistants, physiotherapists, anesthesiologists, radiology technicians, and ward supervisors). Inclusion criteria were: HPs who had obtained professional qualification certificates and agreed to participate in the study. Exclusion criteria were retired HPs, refresher HPs, and student HPs; HPS with less than 6 months of work experience; or HPs missing more than one month of work due to illness, marriage, maternity, or other reasons. Chronotype of participants was determined through the Horne-Ostberg questionnaire (HOQ). Evaluation of WRQOL was obtained using the “Work-related quality of life-2” (WRQOL-2) scale and its subscales.

**Results:**

We included 106 participants from 22 departments. Sex-ratio was 1.2 and median age was 33.5 years (IQR=15). Nurses represented 84.5% of participants. Median WRQOL-2 in our cohort was 3.25 (IQR=0.92) with low to very-low WRQOL accounting for 30.2% of cases (n=32). Participants with an evening type chronotype had significantly lower total WRQOL-2 score compared to morning type (median=2.8 (IQR=0.8) vs 3.7 (IQR=1.2), p=0.002). A negative correlation was found between morning type participants and WRWOL-2 subscores, including job career satisfaction (p=0.044) and stress at work (p=0.049).

**Conclusions:**

Our study found that evening-type healthcare professionals reported significantly lower work-related quality of life (WRQoL-2) scores compared to morning types, aligning with previous research. Misalignment between chronotype and work schedules can lead to increased strain and reduced job satisfaction. Negative correlations between morningness and WRQoL-2 subscores were observed, consistent with findings by Honkalampi et al. (2022).

These results underscore the importance of considering chronotype in scheduling to optimize healthcare workers’ well-being and performance.

**Disclosure of Interest:**

None Declared

